# Supplementary figures and images for: Collective intelligence defines biological functions in Wikipedia as communities in the hidden protein connection network
Source: PLoS Comput Biol. 2020 Feb 18;16(2):e1007652. doi: 10.1371/journal.pcbi.1007652 (PMC7048313; doi:10.1371/journal.pcbi.1007652)

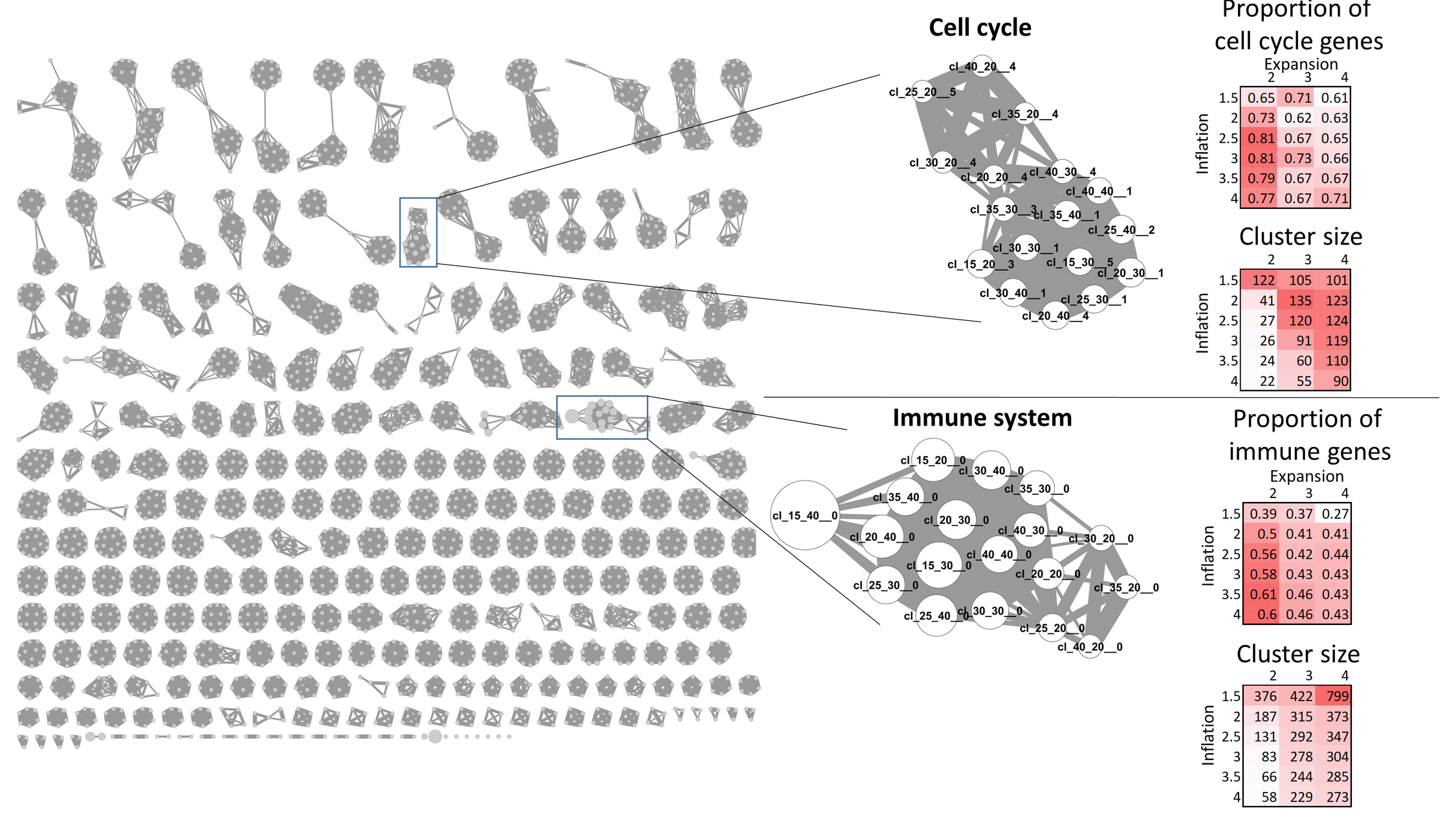

Supplement: S1 Fig — Clustering of the graph of hidden connections between proteins through WikiPedia network was repeated 18 times for various combinations of inflation and expansion parameters, including the default one (inflation = 2, expansion = 2). For the application of each MCL run, a list of clusters larger than 4 proteins was ranked by size. On the left, graph shows intersections between the clusters obtained using the multiple runs of the MCL algorithm. Each node in the graph represents a cluster, labeled in the form ‘cl_(Inflation)_(Expansion)_(Cluster Number)’ (smaller cluster numbers corresponds to the largest clusters, the numbering starts from zero). The node size is proportional to the size of the cluster. The edges in the graph represent intersections between the clusters characterized by Jaccard similarity index larger than 0.4. The width of the edge is proportional to the value of Jaccard similarity index. On the right, zooming on two groups of clusters is shown, corresponding to the communities “Immune system” and “Cell cycle” from Fig 3. Each combination of Inflation/Expansion parameters in each group is characterized by the proportion of cell cycle genes (624 genes from REACTOME Cell cycle pathway) and immune-related genes (1170 genes from GO ‘Regulation of immune process’) correspondingly, and the cluster size. (TIF) [file pcbi.1007652.s001.tif]

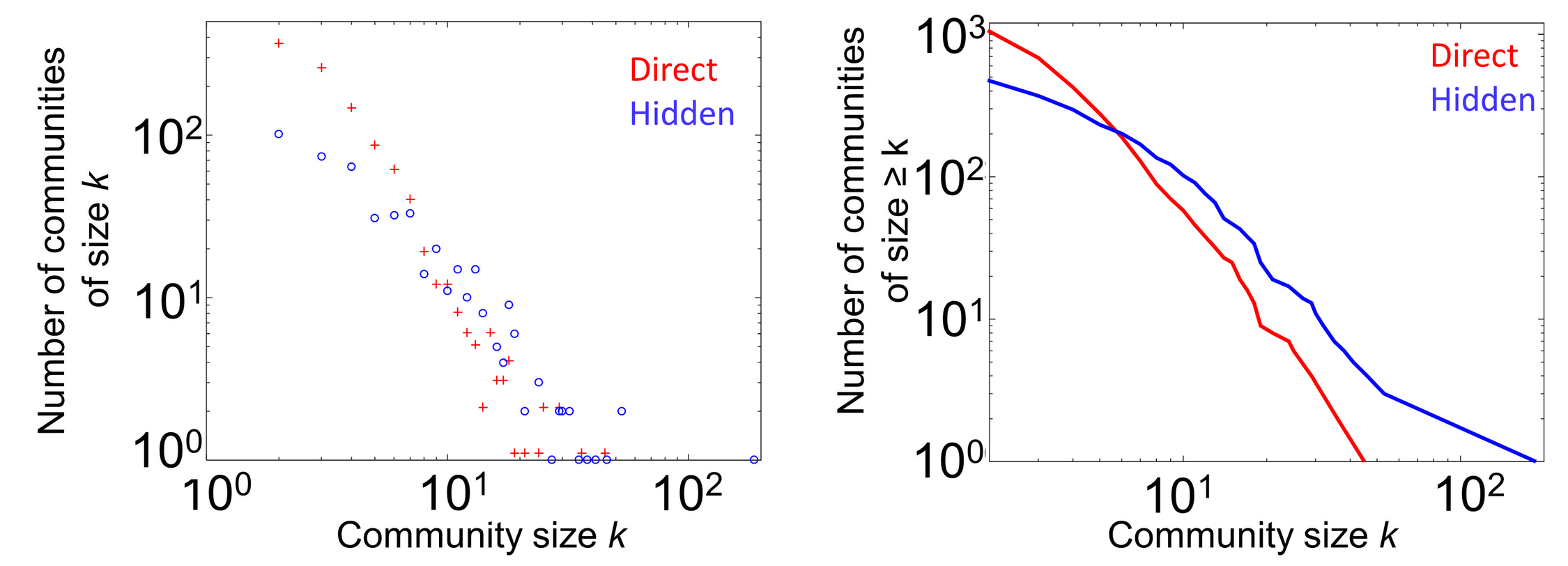

Supplement: S2 Fig — The MCL custering algorithm was applied with the same parameters to the largest connected components of both networks. Community size distribution is shown in the left panel. Cumulative number of the communities larger than a certain size is shown in the right panel. (TIF) [file pcbi.1007652.s002.tif]
