# Supplementary material for: Collective intelligence defines biological functions in Wikipedia as communities in the hidden protein connection network
Source: PLoS Comput Biol. 2020 Feb 18;16(2):e1007652. doi: 10.1371/journal.pcbi.1007652 (PMC7048313; doi:10.1371/journal.pcbi.1007652)
Supplement: Attachment — Submitted filename: Answer2Reviewers.docx [file pcbi.1007652.s005.docx]

**Reviewer #1**

When defining the hidden communities by graph clustering, the authors use Markov clustering with default hyper-parameters. In many cases, the results of clustering is very sensitive to the setting of hyper-parameters, did the authors try other combinations of setting and did the results look quite different? The current results consist of more than 250 communities, which could be reduced for more concise visualization.

This is an important point. Of course, the distribution of sizes and precise composition of graph clusters will inevitably (this is not specific for this particular study) depend on the precise parameter values chosen for Markov clustering. Therefore, we have investigated the effect of changing Markov clustering parameters on the number and composition of the communities in the hidden protein connection network. The results are briefly reported in the manuscript. Our conclusion was that the clusters produced with different parameter values can be unambiguously matched to each other in most of the cases (however, for smaller clusters we can observe splitting or merging the clusters). This means that at least qualitatively the conclusions reported in the manuscript do not depend strongly on the chosen values of clustering parameters. Moreover, we investigated the biological significance of two groups of clusters containing community “Immune system” and “Cell cycle” identified previously with default MCL parameters. We observed that increasing Expansion parameter is usually detrimental for the biological significance of the cluster, while increasing Inflation improves the significance. However, increasing values of Inflation tends also to produce smaller clusters. From our analysis we concluded that the default combination of parameters represents a good balance between biological significance and the cluster size.

Concretely, we introduced a new Supplementary Figure 1 “” and introduced an additional explanation in the manuscript:

*“Parameter analysis of the MCL algorithm application to the graph of hidden protein connections has been performed (Supplementary Figure 1). We showed that the clusters in the hidden protein connection graph can be easily matched for different reasonable combinations of MCL parameters. This means that most of the conclusions reported in this study do not qualitatively depend on the exact choice of the clustering parameters (even though the number of detected communities and their sizes can vary). Moreover, it is shown that the default combination of parameters represents a good balance between the biological significance and the size of the produced cluster.”*

In Line 227 and Fig. 4A, the authors asserted that the hidden links changed more the the direct links through evolution, this is understandable because the polyline connections are more fragile. What may be more interesting is what changed in the direct connections (4%). Did these reflect alteration of knowledge or findings? I notice that some of the connections in 2013 does not exist in 2017, does this have any particular meaning?

We agree with the reviewer’s argument on more fragile polyline connections. However, in the new version of the manuscript we introduced an additional study (see our answer to Reviewer 3) which also shows that the pages explaining indirect connections (‘associated pages’) are edited significantly more intensively than the protein pages themselves, and than a random WikiPedia page.

The reviewer points to an interesting observation on the evolution of direct connections. Indeed, the 2017 Wikipedia snapshot contains 14% more direct links compared to 2013 version. At the same time 3% of direct links present in 2013 version has disappeared in 2017.

First, we looked in more detail on the dynamics of direct connections between 2013 and 2017 snapshots of Wikipedia. We manually inspected the history of few tens of pages-sources of the links which were removed from the network of direct interactions in 2017. Our conclusion was that the main reason for this was a technical artefact. At the initial stage of Gene Wiki project, automated Protein Box Bot included hidden comments imported from standard protein annotations, and most of the protein pages in 2013 contained this automated box with automatically inserted hyperlinks to other protein pages (which could be erroneous and updated further). In the period starting from 2016-2017, there was a massive effort to transfer these automated summaries into the main body of the article. During this process, either certain links were not included in the main body at once or they were not properly hyperlinked. Most of these inconsistencies have been corrected afterwards, and most of the links missing in 2017 were recovered later in 2018 or 2019. Therefore, the observed removal of direct links between protein pages, were due to a transitory technical artefact which was difficult to control for the crawler. However, its effect was relatively minor.

We also reconstructed a network of direct links between protein pages appeared in 2017 WikiPedian snapshot. The set of 100 most connected proteins in this network was enriched in the GO categories similar to what is already reported in the manuscript in the paragraph starting from “We verified if these proteins were enriched in a particular biological function. To answer this question, 181 proteins whose PageRank improved more than two-fold were tested for enrichment in reference gene sets using ToppGene.” Indeed, this is yet another indication of increased activity of curation efforts in WikiPedia Gene Wiki project related to certain specific fields.

In order to take into account this reviewer’s remark, we included the following sentence in the text:

*“Of note, part of the changes in the direct protein links were caused by the process of translating the automated annotations produced by Protein Box Bot into human-curated article texts. For example, this explains the disappearance of a part of direct links in 2017 compared to 2013.”*

**Reviewer #2**:

a) Throughout the paper the authors refer to node degree as `node connectivity`, if I am not mistaken. I would suggest replacing node connectivity by node degree wherever applicable, since node degree is a more popular way to refer to the number of neighbors (as well as to avoid confusion with graph connectivity [the minimum number of elements (nodes or edges) that need to be removed to separate the remaining nodes into isolated subgraphs].

We agree with this remarks and changed the term ‘node connectivity’ to ‘node degree’ systematically throughout the text. We left, however, the term neighbourhood connectivity as a standard name for the measure of connectedness of a group of nodes.

b) Page 3, lines 118-124.

1) Do we understand why the overlap between Wikipedia and databases is so low?

First of all, we should notice that the overlap in terms of the absolute number of common edges is quite low when any two independently built pathway databases are compared. More coarse-grained characteristics of interaction networks such as node degree distribution are relatively well conserved (similarly to what we show in Figure 1D). This is relatively well-established fact which reflects the paucity of our knowledge about biological networks, as well as context-specificity of many links, also used semantics, quality criteria, etc. For example, we made a simple computational experiment and selected a random sample of interactions from BIOGRID database of the same length as the number of direct protein links in Wikipedia (18000), Each interaction in this set was between genes contained in the WikiPedia network. We repeated this random selection 100 times. Then, on average, the intersection between this random sample and SIGNOR set of links was 290 links (less than 5% of total SIGNOR interactions). At the same time, the intersection between WikiPedia network of direct interactions with SPRING is 861 links (21% of SPRING links). This calculation demonstrates that the intersection between direct links and SPRING is not that small relative to what we could expect by comparing two interaction databases (e.g., SPRING and BIOGRID).

As we argue in the manuscript, the overlap is highly significant but this is not surprising given that the set of direct connections was initially fetched from the high-confident interactions described in the BioGrid database.

We introduced a short discussion on the absolute number of overlapping links in the manuscript. In particular, we introduced two additional sentences:

*Also, the intersection size between the set of direct links and existing pathway databases is small in absolute numbers, but it is high relative to expected intersection between independently built pathways databases in terms of the number of common links. The intersection between hidden links and pathways databases is expected to be smaller, because hidden links are not supposed to describe physical interactions but rather reflect the indirect functional proximity between proteins.*

*This overlap is, however, not completely surprising, given that a number of direct interactions between proteins were automatically imported from BIOGRID pathway database (for example, around 60\% of direct links between protein pages are also found in BIOGRID).*

2) Direct and hidden links are not mutually exclusive, is it correct? Then how to understand 861 direct and 170 hidden links matched to SIGNOR? 861 direct and 170 hidden which are not direct? Or some of the 861 direct might be hidden and vice versa?

It is indeed correct and valuable remark, and we thank the reviewer for noticing this. Direct and hidden links are not mutually exclusive even though their meaning is quite different. Having a direct link means having a hyperlink between two protein pages. If at the same time two pages are connected by a hidden link then it means they are also closely connected by indirect paths.

On average, approximately 10% of direct links are also hidden links. However, when one compares hidden interactions to a database of physical interactions, this might create a bias towards simultaneously direct+hidden interactions. This indeed was the case, and among 170 hidden interactions common between Wikipedia and SIGNOR there were 105 direct+hidden interactions (majority).

In order to make this clear, we modified the following sentences in the manuscript:

*Of note, on average 10\% of direct protein links are also contained in the hidden protein link network, i.e. they are 'direct+hidden' links. However, in this comparison, there was a strong bias and among 170 matched hidden links there were 105 'direct+hidden' links. For Pathway Commons, we found 4768 proteins in common with Wikipedia protein page list. These proteins were connected in Pathway Commons by 269665 interactions from which we found 8212 direct and 2563 hidden links (of which 795 'direct+hidden') matched in Wikipedia.*

This correction, however, does not change any of our conclusions.

3) It seems that metrics like precision and recall would be more adequate than Fisher test against the null hypothesis that any two protein can be connected.

We quantified precision and recall for the intersection between wikipedia links and pathway databases, assuming that Wikipedia links is a set of ‘predicted links’ and the pathway database contains ‘true links’. For reference, we also used SIGNOR as a set of ‘predicted links’. In all comparisons, we restricted the links only to a set of genes common between two compared sets. The result looks like this table:

However, we think that referring to precision and recall in this comparison might mislead the reader of the manuscript. The aim of analysing links in WikiPedia is not to ‘predict’ interactions between proteins. Also, nobody can claim that the interactions existing in pathway databases represent ‘true interactions’ or include a complete set of them. Therefore, we do believe that a ‘symmetrical’ comparison between two sets for their overlap (Fisher exact test) characterized by odds ratio is more appropriate here. However, by themselves these numbers do not mean much. We can make a kind of trivial conclusion that it seems that Wikipedia interactions are largely contained in BIOGRID (which is known from the history of Gene Wiki project). All odds ratio are highly significant in all comparisons but still not very high in terms of absolute intersections between links, which is true in all comparisons between pathway databases and Wikipedia. In other words, a set of Wikipedia links is in many aspects similar to publicly available pathway databases. Stating this was the only meaning of making comparison with other pathway databases.

c) Page 6, line 267. Please provide the details of the web-crawler and possibly the code public.

We are sorry for an incomplete sentence in the submitted manuscript. Now we clarify this statement with the following:

“Global network of links between English Wikipedia pages was extracted using in-house web crawler, for 2013 and 2017 years. This procedure creates a link from Wikipedia article A to article B when there is at least one citation of article B in the text of article A (see details in [11])”.

Concerning the availability of the crawler, it seems that starting from a few months ago, there is no more need of using it since the whole content of WikiPedia, including the links table, is provided online in the form of dumps. For example, all the internal links and titles of WikiPedia pages can be directly downloaded from “<https://dumps.wikimedia.org/enwiki/>”, file “[enwiki-latest-pagelinks.sql.gz](https://dumps.wikimedia.org/enwiki/latest/enwiki-latest-pagelinks.sql.gz)”. Because of this, we do not think that providing the code of our in-house WikiPedia crawler is now meaningful.

**Reviewer #3**

The manuscript is generally well written although some sentences appear rushed (see line 267 for an example).

We apologize for an incomplete sentence in the submitted manuscript. Now it is completed with the following:

“Global network of links between English Wikipedia pages was extracted using in-house web crawler, for 2013 and 2017 years. This procedure creates a link from Wikipedia article A to article B when there is at least one citation of article B in the text of article A (see details in [11])”

A major drawback of the manuscript is the lack of an articulated problem or question to be solved: the line of argument is that proteins and genes on Wikipedia can be represented as a directed weighted network, and that network methods can be applied to study the network. What is missing is a goal, however tentative, that would give a direction of both the narrative and the computations. Such a goal would also help biologists comprehend why they should be interested in this description.

We made an effort to clarify the motivation behind this study and justification of the methodological choices.

Choices of design, methods, and measures often receive little to no justification. For example, no clear justification is given to why only two snapshot versions of Wikipedia are considered, and why the focus is on versions of 2013 and 2017 (and whether/how those choices can impact the results).

The 2017 snapshot of WikiPedia was used because it roughly matched 10 years from the start of the Gene Wiki project. The 2013 snapshot roughly corresponds to the start of the activity or users to actively re-write protein pages in WikiPedia (modifying them from the automatically generated summaries). Generating historical snapshots of WikiPedia remains a time-demanding task significantly loading the WikiPedia API, so we reused two snapshots that we had already in our possession. We note that after submission of the manuscript, a new mechanism for storing the complete WikiPedia dump, including complete link structure, has been introduced, so such analyses as in this study will become easier to do in the future, with higher temporal resolution.

We clarified this aspect in the manuscript by stating:

*We used these two snapshots because year 2017 signifies approximately a decade of existence of the Gene Wiki project, and year 2013 signifies the beginning of an active curation effort on the protein pages after the initial automated bot-based page and hyperlink insertion phase. Of note, generating historical snapshots of WikiPedia remains a time-demanding task significantly loading the WikiPedia API, so we reused two snapshots that we had already in our possession.*

Choices are not unreasonable (e.g., PageRank, the community detection algorithm, Jaccard index for pairwise cluster intersection) but there are still many alternatives, and within those alternatives different parameters could be selected, all of which would affect the descriptive statistics.

We totally agree with the Reviewer that there exist alternative methods to answer the questions posed in this study. However, reduced Google Matrix remains the only method which can quantify indirect connections in a large (5M nodes) oriented graph, taking into account the whole graph structure. Using PageRank is tightly related to the use of the reduced Google matrix approach. Using Jaccard similarity is a standard choice to quantify the intersection between two sets (this choice does not imply use of any parameters).

For the community detection algorithm, Markov clustering is the standard choice in the analysis of biological networks, for the following reasons:

1. MCL applied to PPI networks has been shown to be significantly tolerant to noise and behaves robustly (https://bmcbioinformatics.biomedcentral.com/articles/10.1186/1471-2105-10-99)
2. The MCL paper has 433 citations
3. Examples of papers using it:
   a. <https://www.nature.com/articles/nm.3967>
   b. <https://www.nature.com/articles/ncb2549>
   c. <https://genomebiology.biomedcentral.com/articles/10.1186/gb-2012-13-12-r112>
   d. https://www.sciencedirect.com/science/article/pii/S2211124713004695

For example, community size is known to depend on the resolution of the algorithm used to discover those communities, so one needs to evaluate communities across multiple sets of parameters to be able to convincingly show that community size is stable across parameters and is therefore a feature of the network, not the algorithm.

We agree with this argument, and therefore, conducted a sensitivity analysis with respect to the choice of parameters of network clustering algorithm demonstrating that existence of the communities is a robust conclusion (see also response to Reviewer #1 and a new Supplementary Figure 1).

However, we note that the community size even theoretically can not be stable with the change of the main MCL parameters. Larger values of inflation parameter produces smaller clusters, this is an intrinsic feature of MCL. This would be unavoidable in any network clustering algorithm. However, we show that whatever the reasonable choice of the parameters is, the produced communities can be matched to each other in the absolute majority of the clusterings. This is a good feature of clustering and a confirmation of network modularity highlighted in Figure 3. This means that most of the qualitative conclusions made in the study are not dependent on the choice of MCL parameters. In order to determine the optimal parameter choice, one needs to use some other properties of clusters which do not depend on the clustering itself: for example, it can be the biological significance of the clusters. In our additional analysis, we verified that the default choice of parameter for MCL corresponds to a good balance between community sizes and the expected biological significance of the communities.

The main conclusions appear suggestive but not fully supported. For example, the authors concluded:

"The most striking conclusion from this analysis is the existence of pronounced small-scale (10-20 proteins on average) clusters (communities) in the network of hidden protein connections. The absolute majority of these clusters have rather clear biological meaning which was quantified by the functional enrichment analysis. This is in contrast with the previous conclusions about the power law-like distribution of connectivity of protein pages in the global Wikipedia network.” Unfortunately, no frequency distribution plot appears to show the number of clusters with a particular size. Only average values appear to be provided. The authors report clear outliers—clusters (in the networks of hidden protein connections) with size of 150 or more proteins in the Wikipedia versions of 2013 and 2017 (see line 173). The size of those largest communities in 2013 and 2017 does not rule out the possibility of a heavy-tailed distribution. Further, if the power-low argument is considered essential, a more informed approach would be to directly evaluate whether the distribution is power law-like or not rather than to infer it from the mean number of communities. Further, in the presence of outliers, the median (instead of the mean) would likely give a more realistic estimate of the average community size.

We agree with this remark. Now we compare the distribution of community sizes explicitly, both hidden and direct networks in newly introduced Supplementary Figure 1. This comparison clearly shows that, with the same parameters of MCL clustering algorithm, the hidden link network is characterized by systematically less numerous small communities (of size < 8) and more numerous large communities (of size >=8), and that the distribution of community sizes for the hidden link network clearly deviates from the hypothetical power law distribution (small communities are under-represented, the heavy tail is cut out). We should note that this analysis is complementary to the previously reported Fig2B,C and D aiming at comparing the hidden and direct connection networks before detecting their communities. Also, of note that the power law-like distribution of protein page connectivity in the global Wikipedia network was first reported in Huss, 2008 (Figure 2C).

Furthermore, we now mention both mean and median community sizes in the Results text.

We do not think that the rigorous testing of the power law is essential in this context.

We added several sentences to the main body of the text, Results section:

*For comparison, We also applied MCL algorithm with the same default parameters to the network of direct connections (Supplementary Figure 1). In agreement with the results described above, the community size distribution was different for the networks of direct and hidden connections. It reminded the power law distribution in the case of direct connections, while in the case of clustering the network of hidden connections, the tail of small size communities was much shorter than it would be expected from the power law.*

An association between community structure and biological function is strongly postulated but is only tentatively supported by suggestive examples. See for example lines 331–333: "Existence of such clusters (communities) points out to emergence, due to collective intelligence of Wikipedia editors, of relatively well defined groups of proteins sharing the common biological function (such as cell cycle), structural feature (such as SH2/SH3 domain) or other common topics.

We would like to point to the paragraph starting with “Using HUGO symbols matched to the Wikipedia protein page titles, we performed function enrichment analysis”. From that point, we describe a systematic test on the association between communities in WikiPedia and known biological function definitions (such as GO terms). The results are quite detailed and reported online here: <http://www.quantware.ups-tlse.fr/QWLIB/wikiproteinnets/topgeneanalysis/index.html>.

We clarified in the text that the association between biological functions and the community composition was systematically tested by modifying the above-cite phrase:

*Existence of such clusters (communities) points out to emergence, due to collective intelligence of Wikipedia editors, of relatively well defined groups of proteins sharing the common biological function (such as cell cycle), structural feature (such as SH2/SH3 domain) or other common topics, as it was confirmed in this study by the systematic functional enrichment analysis.*

A major question is whether the clusters/communities have emerged due to collective intelligence or due to a few very active contributors, which are often considered to be a defining feature of Wikipedia. Essentially, the paper has not examined the distribution of editors producing the connections between pages, and has not ruled out the hypothesis that the clustering of proteins is due to a few very active editors that formed direct and/or indirect connections between many pages. Thus, a key property of the gene Wikipedia network noted in the title—collective intelligence—is assumed to be true but is not directly examined.

We agree with this remark. Indeed, the critics of WikiPedia is often in that it is a “Wisdom of few” rather than “Wisdom of crowds” (<https://arxiv.org/abs/1604.03044>). We now briefly mention this discussion in the Introduction.

In order to analyse this aspect in detail, we made an effort to estimate the number of contributors involved in curating protein pages and associated pages and now report these results in the manuscript. We introduced a new section “Analysing WikiPedia contributor set defining direct and indirect protein connections” and a new Figure 5 illustrating the conclusions from this study. Briefly, we estimate the number of active expert contributors working specifically on the protein and associated pages to approximately 900 people, and we can conclude that these contributors mostly have biological or scientific expertise. The distribution of contributor involvement follows the power law-like distribution, with few hub contributors with record number of contributions to protein or associated pages. However, these hub contributors explain only 8% of total edits to associated pages and 30% of total edits in protein pages. The median number of expert contributors per protein page is 3 while it is 9 for an associated page, which can be compared to 7 median contributors per random WikiPedia page.

Altogether, this shows that the effort for curating protein pages is indeed rather dominated by few contributors. However, the set of associated pages, defining the indirect links between proteins, is curated in a distributed way, by an expert community of approximately 1000 wikipedians, having different levels of expertise and involvement of course, which is a feature of the wisdom of the crowd mechanism.
